# Effect of Infill Density on the Mechanical Properties of Natural Peek Processed by Additive Manufacturing

**DOI:** 10.3390/polym17030347

**Published:** 2025-01-27

**Authors:** Efrén Vázquez-Silva, Jonnathan Andrés Pintado-Pintado, Freddy Patricio Moncayo-Matute, Paúl Bolívar Torres-Jara, Diana Patricia Moya-Loaiza

**Affiliations:** Grupo de Investigación en Nuevos Materiales y Procesos de Transformación (GIMAT), Sede Cuenca, Universidad Politécnica Salesiana, Cuenca 010105, Ecuador; jpintadop1@est.ups.edu.ec (J.A.P.-P.); fmoncayo@ups.edu.ec (F.P.M.-M.); ptorresj@ups.edu.ec (P.B.T.-J.); dmoyal@ups.edu.ec (D.P.M.-L.)

**Keywords:** polyether-ether-ketone (PEEK), additive manufacturing, infill density, mechanical characterization, tensile properties, compressive properties, flexural properties, biomedical applications

## Abstract

In the present investigation, the mechanical properties of natural polyether-ether-ketone (PEEK), processed by additive manufacturing applying fused deposition modeling (FDM) with three different infill densities, are investigated. Mechanical characterization was performed through destructive testing. Specimens were designed in CAD software and printed with controlled infill densities of 40%, 70%, and 100%, using a rectilinear pattern. The results showed that increased infill density improves mechanical strength and stiffness but reduces ductility and energy absorption capacity. For considered infill densities, maximum stress levels reach values of 107.53±6.29MPa, 114.32±11.95MPa, and 63.96±2.39MPa, respectively, against compression, bending, and tensile loading. These findings offer crucial information for optimizing infill density in manufacturing high-strength components for industrial and biomedical applications. As a result, practical guidelines are provided for the design of medical devices, such as implants, achieving an appropriate balance between mechanical performance and material efficiency.

## 1. Introduction

Industrial applications of polymeric materials as more economical and environmentally friendly alternatives are growing sustainably. In the specific case of polyether-ether-ketone (PEEK), it is applied in industry (see, for example, [1,2,3]) and in medicine (see, for example, [4,5,6,7]).

In [8], a classification of the mechanical properties of PEEK family polymers was proposed and a comparison was made with other polymers to determine their versatility in biomedical applications. The authors reported a maximum tensile stress for medical grade PEEK-OPTIMA, without specifying how the mechanical tests were performed.

Three-dimensional-printed PEEK exhibits excellent flexural and tensile strength. In [9], the authors found that changes in temperature and printing speed affect the material mechanical properties. Additionally, the bio-inert nature of PEEK can make adhesive bonding difficult. Options are also offered to improve bond strength. A comprehensive overview of the research progress on the mechanical properties of PEEK for dental applications is provided.

In [10], standard PEEK parts were 3D printed by the fused deposition modeling (FDM) method for bending and compression tests. Nozzle diameter, nozzle temperature, and printing speed were discussed. The density and dimensional accuracy of the printed parts were evaluated according to ISO standard 178 for three-point bending test specimens in the shape of small bars and ISO standard 604 for the compression tests of cylindrical specimens.

In [11], FDM was employed to fabricate PEEK samples for performing tensile tests to investigate the relationship between various thermal processing conditions in this process, such that the raster angle, nozzle temperature, ambient temperature, post-heat treatment temperature after FDM, and the mechanical properties of pure PEEK material. In the study, the mechanical properties of the printing filament were assumed according to ISO 527 [12] regulations; the tested specimens were not clarified either.

In a systematic review by Moby [13], optimal printing parameters were established for FDM 3D-printed PEEK elements with mechanical properties suitable for dental restorations. The selected studies were difficult to compare due to the variability of the printing parameters and the types of PEEK. It seems interesting to use a high infill rate, a high chamber temperature close to that of the printing temperature, and a heat post-treatment to obtain 3D PEEK elements presenting properties adapted to use as dental restorations.

Article [14] analyzed the use of PEEK as a printing material to explore the combined effect of multiple factors: different printing temperatures, printing directions, printing routes, and layer thicknesses. The work investigated how these factors influence the tensile strength, flexural strength, crystallinity, and grain size of printed FDM pieces. The goal was to attain results comparable to those achieved through injection molding. Among the main findings, it was observed that the greater the tensile strength of the printed sample, the greater the uniformity in the grain size and the greater the crystallinity of the material.

Some of the remaining challenges to successfully performing FDM of PEEK are addressed in [15]. In the research, finite element analysis (FEA) was applied to simulate the melting conditions and fluidity of the polymer in a flow channel to establish the necessary parameters to achieve prints with good surface quality and better mechanical properties. FDM experiments were conducted to investigate the effects of printing temperature, speed, and layer thickness on the mechanical properties, microstructure, and surface quality of printed parts. It is suggested that a heating temperature higher than 440 °C, a printing speed of 20 mm/s, and a printing layer thickness of 0.1 can improve the density of PEEK parts, reduce internal defects, and strengthen the bond between the printed layers and the filling filament. The tensile strength was measured at room temperature and the specimens were prepared according to the tensile test method and GB/T 1040-92 standard [16].

In another study, Wu et al. investigated how layer thickness and raster angle affect the mechanical properties of PEEK 3D-printed parts and found that a layer thickness of 300μm and a raster angle of $0^{\circ }/90^{\circ }$ offer the optimal properties for certain industrial applications [17]. However, this study does not specifically address how infill density, one of the key parameters in FDM, affects the mechanical properties of PEEK.

On the other hand, Liu et al. studied the dynamic mechanical and thermomechanical properties of FDM-processed PEEK, evaluating the material behavior under tensile loading and observing the fracture morphology. Their results show a direct relationship between the printing process and the fracture morphology, but they also omit the specific impact of the infill density on the mechanical properties, a crucial aspect for material optimization in applications [18].

In [19], it was demonstrated that triangular and cubic infill patterns can improve the stiffness and strength of 3D-printed parts. Exploring these patterns in combination with various infill percentages could offer more effective solutions for specific applications.

Several results have been published on the influence of printing patterns or infill densities when using other polymeric materials or manufacturing techniques, such as Fused Filament Fabrication (FFF). For example, in [20], the authors evaluated the effects of parameters such as nozzle temperature, platform temperature, infill percentage, layer height, and printing speed, during the FFF process, on the mechanical and tribological properties of PEEK. The variation of surface mechanical properties was studied, with indentation and roughness tests, and bulk mechanical properties, with the help of tensile tests. Another study [21] focused on the dynamic mechanical properties of FFF-printed PEEK with various infill patterns: line, cubic, hexagonal, and grid. The authors attempted to understand the failures in each pattern with the help of finite element analysis (FEA). The results demonstrate notable differences in performance characteristics depending on the infill patterns. On the other hand, in [22], the impact resistance and energy absorption behavior of thermoplastic polymer composites under quasi-static compression was investigated. The authors considered four design patterns: cuboctahedron, Kelvin cell, square-truncated cube, and square dividend geometry. FDM manufactured the specimens, and the structures were fabricated with four filamentary materials based on different thermoplastic polymers. The influence of the composite on the impact resistance characteristics was studied experimentally, obtaining the most interesting results for carbon fiber-reinforced polylactic acid (PLA-CF) and carbon fiber-reinforced glycol-modified polyethylene terephthalate (PETG-CF). Also, in [23], the effect of the pattern and the infill density of cubes under the action of a quasi-static axial compression load was investigated. The specimens were manufactured by 3D printing with two different materials: cubes made entirely of PLA, and PLA mixed with carbon fiber (PLA/CF) at a ratio of 70–30% of the total volume. In addition, four filling patterns were considered: triangular, rectilinear, linear, and honeycomb with 20%,40%,60%, and 80% infill density. Destructive tests allowed examination of the effect of filling pattern, infill density, and material types on the crush failure behavior and energy absorption characteristics. The best results were obtained for the honeycomb filling made entirely of PLA.

Despite these advances, there is a significant gap in the literature regarding the influence of infill density on the mechanical properties of FDM-processed PEEK, especially in terms of its performance in biomedical and industrial applications. Although research has been conducted on the behavior of 3D-printed PEEK, the relationship between infill density and mechanical properties has not been thoroughly described; this constitutes a limitation to the optimization of the design and manufacturing of customized devices that require a balance between mechanical strength, material efficiency, and functionality.

In most of the articles mentioned above (except [15]), the conditions for performing mechanical tests were not specified (taking into account those works in which results on mechanical characterization are reported). There is no clarity on the type of printed parts and specimens being tested or the grade of PEEK used, whether it is related to medical or industrial applications.

PEEK is known for its good performance in terms of weight, high yield, and excellent mechanical properties. This polymer has also been found to outperform its conventional metallic counterparts in terms of lower stress protection and better chemical resistance. These good characteristics make it an appropriate material for implantable applications. The limitations of this thermoplastic are also known, such as the need for surface modifications to improve antibacterial, bioactive, and osseointegration properties suitable for in vivo applications. For instance, this is referenced in [24,25,26,27].

The present research focuses on how the infill density applied in the 3D printing process with the FDM technique can affect the mechanical properties of specimens manufactured with natural PEEK (also known as industrial grade PEEK). This is in consideration of the diverse findings reported in the specialized literature regarding the parameters that need to be controlled in the 3D printing process. The tensile, compression, and bending tests conducted adhere to regulations for studying thermoplastic polymeric materials.

Therefore, it is desirable to verify how the maximum stress levels vary under tensile, bending, and compression loads of natural PEEK under considered conditions and to understand the behavior of the maximum loads before failure occurs.

The obtained results aid in understanding the performance of customized medical devices.

## 2. Materials and Methods

For the development of this research, ASTM standards were applied to ensure the rigor and reproducibility of the mechanical tests carried out on natural PEEK specimens. The selection of these standards was based on the need to characterize the material’s properties in a standardized manner and to ensure the comparability of the results. ASTM D638-14 [28] was used for tensile testing; this standard accurately assesses the strength and modulus of elasticity of polymers under tension, providing critical information on the material’s ability to withstand stretching forces before failing. ASTM D695-15 [29] was applied for compression testing, essential for characterizing PEEK’s strength under compressive loads, particularly in applications where the material is subject to crushing forces. Finally, ASTM D790-10 [30] was applied for bending tests to evaluate the behavior of PEEK against loads that generate bending stresses—a key aspect for applications that require resistance to both deformation and bending.

The material used was natural polyether-ether-ketone 450 (PEEK), a high-performance polymer known for its thermal and mechanical resistance, making it suitable for industrial and biomedical applications. PEEK is a highly unstable material when faced with, for example, temperature changes. For this reason, the parameters shown in Table 1 were kept unchanged to reach the lowest possible variability in the results of the destructive tests with the printed specimens. The specimens were manufactured by additive manufacturing using the fused deposition modeling (FDM) technique; this allows the manufacturing method’s impact on the material’s mechanical properties to be evaluated.

In some studies (for example, [31,32,33]), it has been established that for 3D printing with fused deposition modeling, the infill percentages should be between 20 and 80, because less than 20% results in flimsy parts and more than 50% starts to take too long to print and use too much material. Parts that experience moderate stress benefit from mid-range infill densities (20–50%). This ensures sufficient durability while optimizing material use. Components subjected to significant stress require higher infill densities (50–80%). This improves rigidity and load distribution while balancing weight and print efficiency. Thus, for 3D printing, the FDM technique was used, considering only infill density as a variable, with values of 40% and 70%, which are suitable for applications that have been jointly developed by the GIMAT research group and medical institutions in the city. A solid specimen (100% infill density) was also considered to determine the level of maximum stress it supports. Limitation in the availability of PEEK filament was the main reason for deciding to include only these infill density percentages. Challenges related to the possible anisotropy of the material, or the influence of printing parameters such as temperature, speed, and layer orientation on the final properties of the samples obtained, were not taken into account.

Finally, a one-way analysis of variance (ANOVA) is applied to analyze the influence of the infill density of the printed specimens on the maximum stress caused by tensile, compressive, and bending loads.

### 2.1. Mechanical Properties of Natural PEEK

The material used for the study is natural PEEK from Apium Additive Technologies, a high-performance semi-crystalline polymer with excellent mechanical, thermal, and chemical properties. PEEK is widely used in industrial and medical applications for its ability to withstand extreme conditions. The mechanical properties of natural PEEK, specifically in the form of filament roll for additive manufacturing, are highly dependent on the printing process, including factors such as extrusion temperature, layer orientation, and cooling conditions. The mechanical properties of natural PEEK in filament form at 23 °C are presented in Table 2.

### 2.2. Manufacturing Process

A rectilinear infill pattern of 40%,70%, and 100% was used in 3D printing because this type of infill provides a uniform distribution of forces along the structure and guarantees an internal arrangement that maximizes the rigidity and strength of the specimens. The rectilinear pattern offers an alignment of the fibers that improves the transmission of the applied loads and avoids stress concentrations that could generate premature failures. It also facilitates the reproducibility of the results, since the observed behavior is close to that of a solid material. Such a pattern also contributes to reducing the variables in the analysis of mechanical behavior, ensuring that the results depend mainly on the intrinsic properties of the material and not on the complexity of the internal design.

For manufacturing, an INTAMSYS Funmat PRO 410 was used, a high-performance 3D printer specially designed for printing with specific materials at high temperatures: PEEK, polietercetona cetona (PEKK), polyetherimide (ULTEM), and other advanced polymers. This printer is particularly suitable for industrial and research applications, requiring precise high-strength components. Table 1 summarizes the printing parameters used for natural PEEK.

Proper control of the printing environment is essential to ensure the quality and consistency of the manufactured samples. Throughout the printing process of the PEEK specimens, attention was paid to the environmental conditions—temperature and humidity—trying to minimize variations that could affect the properties of the material and, therefore, the results of the mechanical tests.

The printing laboratory, where the INTAMSYS Funmat PRO 410 3D printer was located, was kept at a constant temperature of 22±2 °C, following best practices reported in the literature. For example, ref. [17] highlights that temperature fluctuations can generate defects in the parts, such as deformations or failures in the layer alignment.

Humidity was also strictly controlled, since PEEK filament, as a hygroscopic material, can absorb moisture from the environment, which could modify its viscosity and alter the mechanical properties of the parts. To avoid these problems, the printing environment was kept at a relative humidity below 40%. This process is essential to ensure that PEEK remains in its most stable state [18]. This article reports that residual moisture in the filament can cause premature thermal decomposition, negatively affecting strength and other mechanical properties.

#### Designing of 3D-Printed Specimens

The specimens were manufactured using CAD modeling to ensure adequate dimensional accuracy and compliance with the requirements of the corresponding ASTM standards. The specimens were designed using 3D modeling software, considering the geometric specifications necessary for bending, compression, and tensile tests.

**Figure 1:**
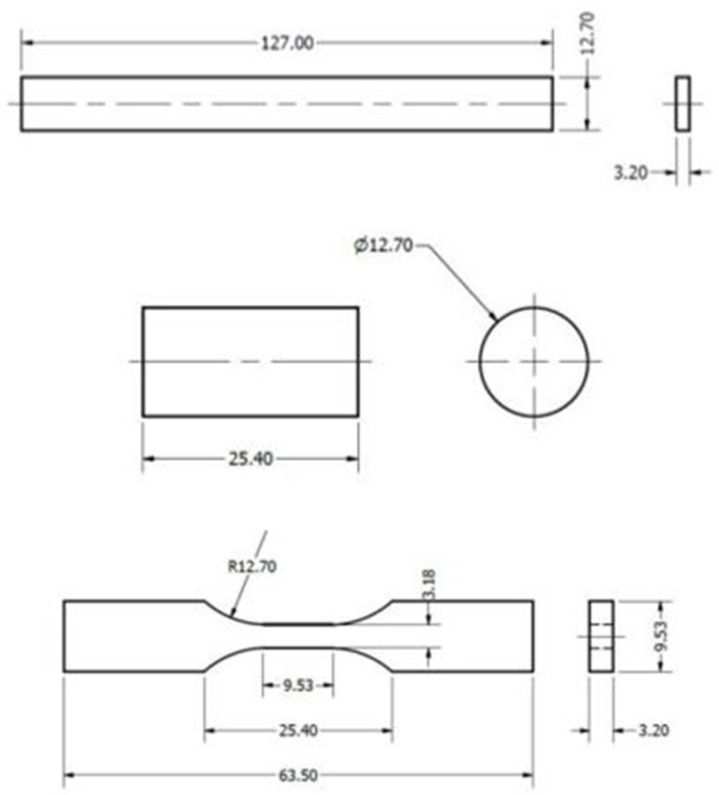
Geometry and dimensions of the specimens for bending test (**top**), compression test (**middle**), and tensile test (**bottom**).

- Flexural Test Specimen (ASTM D790-10) [30]: The CAD model of the flexural test specimen was designed with a length of 127 mm, a width of 12.7 mm, and a thickness of 3.2 mm. This rectangular geometry ensures that, during the test, a homogeneous distribution of stresses is generated along the cross-section, allowing for an accurate analysis of the flexural strength of the material (Figure 1 (top)).
- Compression Test Specimen (ASTM D695-15) [29]: The compression specimen was modeled as a cylinder, with a height of 25.4 mm and a diameter of 12.7 mm. This geometry allows for adequate load stability, minimizing undesirable effects, such as buckling, during testing. The cylindrical design is ideal for ensuring that the load is distributed evenly across the cross-section (Figure 1 (middle)).
- Tensile Test Specimen (ASTM D638-14, Type V) [28]: The CAD model of the tensile specimen was based on the Type V design, which is particularly useful when the amount of material is limited. This specimen has an overall length of 63.5 mm, a width of 9.53 mm at the reduced section, and a thickness of 3.18 mm. The dimensions were carefully defined to ensure that the deformation was concentrated in the reduced section, thus ensuring an accurate characterization of the PEEK properties under tension (Figure 1 (bottom)).

Each CAD model was designed considering specific dimensional tolerances to ensure the quality of additive manufacturing and the reproducibility of mechanical testing. These models were exported to a stereolithography format for the 3D printer and the generation of the G-code.

**Figure 2:**
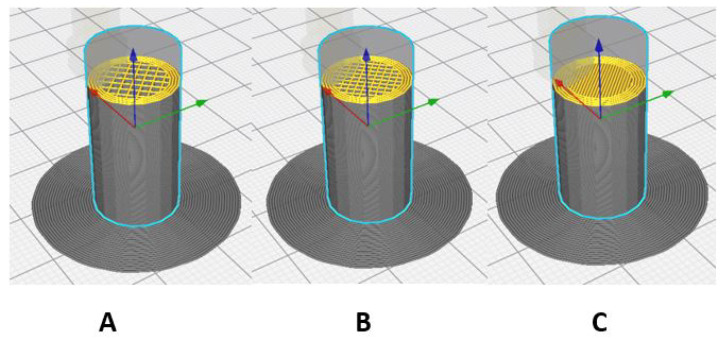
Setting the infill density for compression specimens. (**A**) 40%; (**B**) 70%; (**C**) 100%.

Figure 2 shows the models of the three cylindrical 3D-printed specimens, each with a different rectilinear infill pattern, varying from 40% to 100% density from left to right. These various levels of infill density will affect strength and stiffness, depending on the specific use for which they are intended.

**Figure 3:**
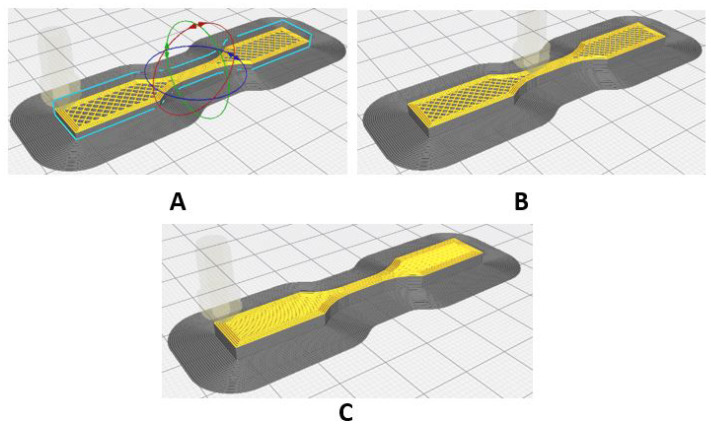
Setting the infill density for tensile specimens. (**A**) 40%; (**B**) 70%; (**C**) 100%.

Figure 3 shows the three V-type specimens for the tensile test, obtained by 3D printing, each with a different rectilinear filling pattern, varying from 40% to 100% infill density from left to right.

This approach seeks to optimize the balance between mechanical strength and weight while ensuring a systematic and controlled comparison of mechanical properties under different densities. A 100% infill simulates a solid material and offers maximum strength, while lower densities allow for reduced material usage and printing time while maintaining a level of strength suitable for applications where weight is a critical factor. Additionally, the use of the rectilinear pattern ensures a uniform internal structure that helps to distribute loads efficiently, avoiding weak points in the specimens. The selected configuration also seeks to validate the effectiveness of additive manufacturing in the production of high-strength components, since PEEK, being a high-quality material for extreme conditions, must be evaluated in terms of its limitations and advantages when manufactured by FDM 3D printing. These configurations will allow identifying the most suitable biomedical applications for each type of configuration, considering factors such as lightness, strength, and functionality of the components produced.

**Figure 4:**
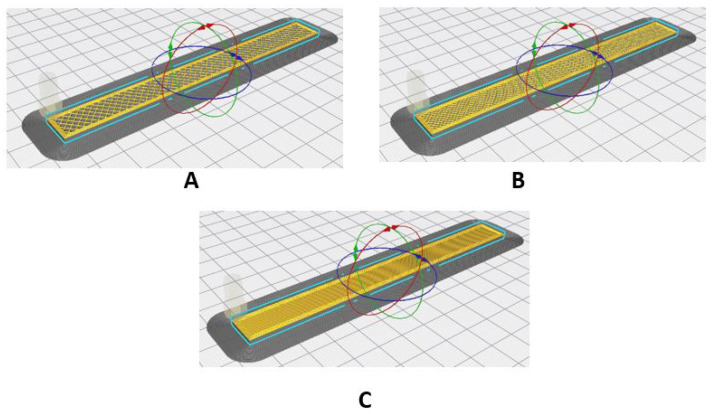
Setting the infill density for flexural specimens. (**A**) 40%; (**B**) 70%; (**C**) 100%.

Finally, Figure 4 shows the configuration of the infill density in the specimens for the flexural tests.

### 2.3. Experimental Setup

To ensure the consistency and reliability of the results obtained in the mechanical tests, some quality controls were implemented throughout the manufacturing process and analysis of the samples. First, a detailed visual inspection of each printed specimen was carried out to ensure that it did not present visible defects such as cracks, air bubbles, or surface imperfections, which could compromise its mechanical properties. Visual inspection is a widely used standard in similar studies [18].

Furthermore, measurements of the dimensions of each sample were made using a digital caliper, verifying that the printed parts met the geometric specifications of the corresponding ASTM standards (D638-14 [28], D695-15 [29], D790-10 [30]). This approach follows the recommendation of, for example, [13], which states that accurate measurements of the dimensions of printed parts are crucial for reproducibility of results and effective comparison of experimental data in additive manufacturing.

In order to validate the consistency of the 3D printing manufacturing process, printing parameters such as extrusion temperature, printing speed, and layer height were controlled, keeping them within the optimal ranges established by the 3D printing equipment manufacturer. This practice is consistent with the methodology described in [17]. This work concludes that variability in these parameters has a direct impact on the quality of the mechanical properties of the printed PEEK.

Finally, post-processing of the PEEK filament was implemented before printing: the roll of filament was placed in an oven and subjected to a temperature of 90 °C for 12 h to eliminate any traces of moisture [17,36].

#### Setting up the Experimental Campaign

The experimental campaign begins with 3D printing of natural PEEK specimens following ASTM standards ([37,38]) and ensuring that the samples are uniform and ready for mechanical testing. Once printed, the test pieces underwent a visual inspection and verification of their dimensions before proceeding with the tests. The Shimadzu machine, equipped with a 20 kN load cell, allows the three types of tests to be carried out—tensile, compression, and bending—changing the tooling accessories as necessary.

The tensile test was performed at a speed of 1 mm/min for 10 min, the compression test at 1.3 mm/min for 6 min, and the bending test at 1.3 mm/min for 8 min. These speeds and time intervals allow for obtaining accurate data on the strength and behavior of the material.

**Figure 5:**
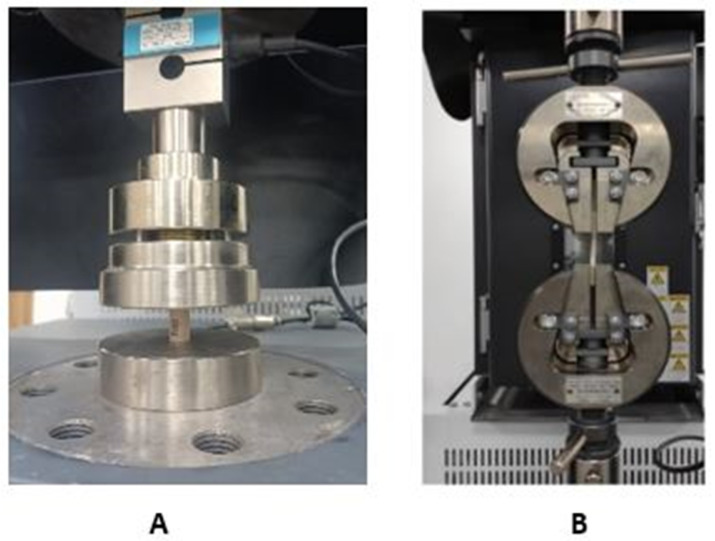

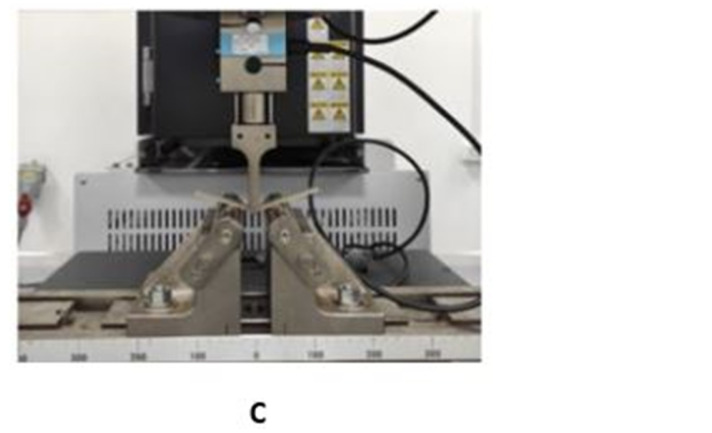
Equipment setup for the experimental campaign and assembly of the test tools: (**A**) compression, (**B**) traction, (**C**) bending.

Figure 5 shows the specimens placed in the respective fixing systems for carrying out the corresponding destructive tests.

## 3. Results and Discussion

### 3.1. Mechanical Force Behavior

#### 3.1.1. Tensile Analysis

**Figure 6:**
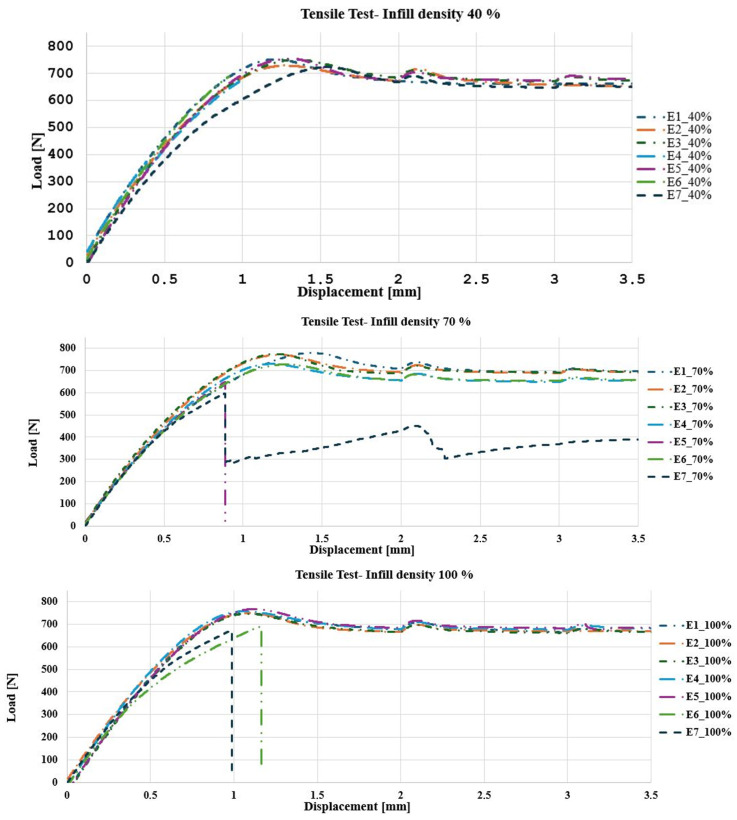
Load [N]–Displacement [mm] behavior. Tensile test: specimens with 40% filling (**top**); specimens with 70% filling (**middle**); specimens with 100% filling (**bottom**).

Tensile tests show how the infill density influences the mechanical behavior. The specimens with 40% filler reach a maximum load of 700±20N, showing ductile behavior. The specimens with 70% filler support a slightly higher load of 750±25N. On the other hand, the specimens with 1000% filler reach a maximum load of 800±30N. As the filler density increases, the specimens become stronger and stiffer. Figure 6 shows the behavior of the load–displacement curves for each infill density.

**Figure 7:**
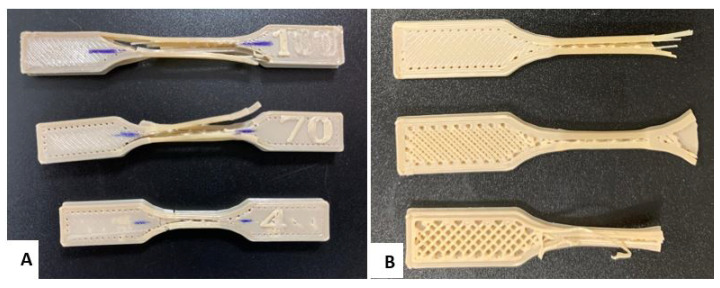
Tensile test specimens: (**A**) Tensile failure of the standardized specimen with filling levels of 40%, 70%, and 100%. (**B**) Cross section of the specimens to visualize the infill density.

In the case of the experimental tensile campaign, the results are as follows: The specimen with 40% filler shows a behavior with a higher elongation (4.85%), a lower modulus of elasticity (2127.04MPa), a maximum stress of 63.34MPa, and a yield stress (yield limit) of 58.87MPa. These results represent the early failure observed in Figure 7A, due to the less dense internal structure that generates greater flexibility and lower resistance to the applied load, which allows the specimen to reach its yield limit more easily.

For the 70% filled specimen, the maximum stress increases to 64.4MPa, the modulus of elasticity rises to 2294.22MPa, and the yield stress decreases to 53.71MPa. These results suggest better internal cohesion and load distribution, resulting in a more uniform failure with a lower deformation capacity before the collapse, indicative of greater strength without reaching extreme rigidity.

Finally, the 100% filled specimen presents a maximum stress of 63.95MPa, a modulus of elasticity of 2436.67MPa, a reduced elongation of 4.27%, and a yield stress of 49.05MPa, which implies a brittle and rigid behavior. This is evidenced by the clean fracture observed in Figure 7A. In [39], it is analyzed how density affects the mechanical properties of PEEK processed by additive manufacturing. The results link density, stiffness, and energy absorption capacity. The authors consider PEEK-CF cellular structures and observe an increase in density and stiffness associated with greater fragility and decreased specific energy absorption due to premature collapse and lower ductility, suggesting that denser structures exhibit more fragile behavior and less efficient energy dissipation. In the present study, PEEK with different infill densities is investigated and shows that higher density improves the stiffness and ultimate strength. Still, it significantly reduces ductility and energy absorption capacity before failure. Samples with 100% infill density exhibit clean fractures and brittle behavior, while those with lower density absorb more energy due to their greater plastic deformation. Both studies agree that increasing density improves strength and stiffness but sacrifices ductility and stress redistribution capacity, leading to abrupt failures. The literature supports these results ([40,41]), which relates high density with increased stiffness and fragility. Therefore, the statement is valid and reflects an intrinsic behavior of PEEK processed by additive manufacturing, highlighting the need to balance stiffness and energy absorption in component design.

The progressive decrease in yield stress with increasing filler density suggests that the material becomes stiffer and less capable of plastic deformation, which favors brittle failure [11]. The rectilinear filler pattern in the cross-section of the specimens (Figure 7B) corroborates the influence of the orientation and density of the material on stress transfer and concentration, which translates into significant differences in strength and ductility behavior [10]. Therefore, as filler density increases, stiffness improves, but deformation capacity and yield stress are reduced, causing higher-density specimens to exhibit more brittle behavior and less tolerance to elongation before failure [9]. Figure 7 shows the specimens with the damage caused by the tensile test.

Table 3 presents a summary of the average mechanical properties with the three different percentages of fillers; seven samples were tested for each percentage.

#### 3.1.2. Flexural Analysis

**Figure 8:**
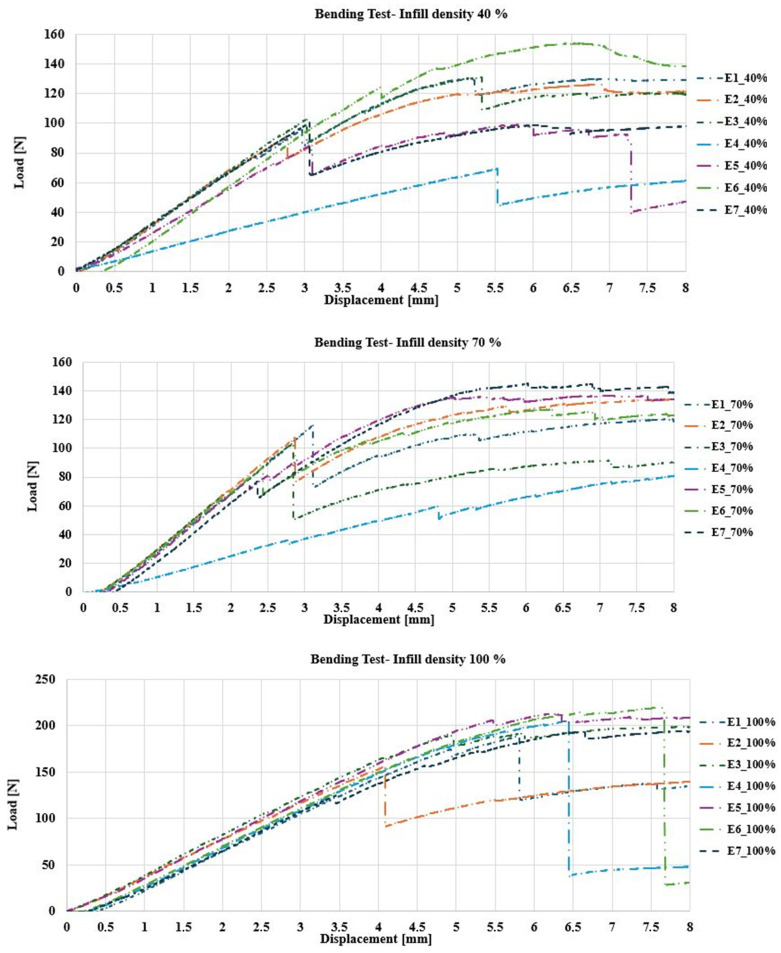
Load [N]–Displacement [mm] behavior. Bending test: specimens with 40% filling (**top**); specimens with 70% filling (**middle**); specimens with 100% filling (**bottom**).

As the density increased from 40% to 100%, a significant increase in load capacity and stiffness was observed, indicating an improvement in the strength of the material. The samples with 100% filler supported an average maximum load of 225.71±11.16N, the samples with 70% filler supported 125.71±11.16N, and the samples with 40% filler reached 102.86±13.32N. These results show that a higher percentage of filler not only contributes to greater strength but also a more uniform and consistent response under load. Figure 8 shows the behavior of the load–displacement curve for each infill density.

The analysis of PEEK specimens under bending load indicates how filler density influences mechanical behavior and failure. The specimen with 40% filler deformed considerably, presenting a marked buckling in the middle part due to the lack of material, which made it more flexible but less resistant, with an approximate deformation of 15–20% before breaking. In contrast, the 70% filled specimen showed an adequate balance between rigidity and flexibility, as it presented a progressive fracture in the center and a deformation in the range of 10–12%, indicating a better stress distribution and greater resistance, compared to the 40% filled specimen. Finally, the 100% filled specimen barely deformed before failing, which demonstrates a very rigid and brittle behavior, with an abrupt fracture and an estimated deformation of 5–8% [9].

**Figure 9:**
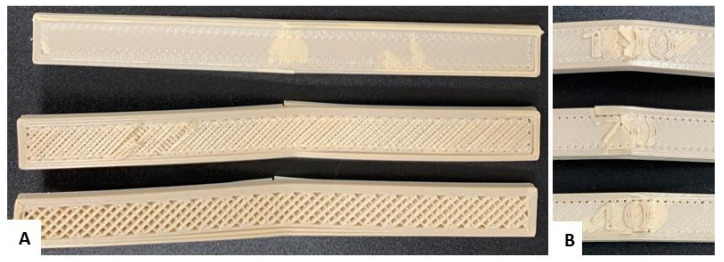
Flexural test specimens: (**A**) Cross section of the specimens to visualize the infill density. (**B**) Flexural failure of the standardized specimen with filling levels of 40%, 70%, and 100%.

This behavior suggests that lower-density specimens are more suitable for applications where energy absorption capacity is important, while higher-density specimens are better for applications where structural stability and strength are essential. These results are consistent with previous studies conducted on composite materials and polymers subjected to bending loading [42,43]. Figure 9 shows the bending test results.

#### 3.1.3. Bending Stresses

The results obtained from the experimental bending campaign on PEEK test pieces with different filler densities demonstrate how, by increasing the filler density (from 40% to 100%), the mechanical properties of the material are significantly altered. The test pieces with 40% filler reached a maximum stress of 63.43MPa, with an elasticity modulus of 2134.71MPa and an elongation of 5.46%. These values show that the structure of the material is less rigid, but with a notable capacity to deform before failure, showing ductile behavior that allows energy to be absorbed at the cost of lower resistance.

By increasing the filler density to 70%, the specimens showed an improvement in flexural strength, reaching a maximum stress of 70.69MPa, along with an elastic modulus of 2676.21MPa and an elongation of 5.54%. This density represents a good balance between rigidity and flexibility, which improves the material’s ability to adapt to the applied load without losing strength. When the filler density reaches 100%, a significant increase in structural strength was observed, reflected as a maximum stress of 114.32MPa and an elastic modulus of 2773.73MPa but with an elongation of 5.46%, which is similar to that resulting from the 40% filler [11].

This behavior shows a highly rigid but also more fragile structure, with an abrupt fracture and minimal deformation before failure due to the high stiffness. Table 4 presents a summary of the average mechanical properties with the three different percentages of fillers. Seven samples were tested for each percentage.

#### 3.1.4. Compression Analysis and Compressive Stresses

The 40% filled specimens reached an average maximum load of 6500±250N, which implies a moderate variability. The lower material density causes the specimens to present more internal heterogeneity, reflected in a significant dispersion in the results. With 70% filled specimens, the average maximum load increased to 7000±200N—that is, with less dispersion than for 40% filled specimens, showing that a greater amount of material contributes to a better consistency in the mechanical response. Finally, the 100% filled specimens reached an average maximum load of 11000±150N, reflecting the greatest uniformity and lowest dispersion of all.

This completely solid structure allowed the specimens to withstand higher loads with very predictable behavior. In general, it was shown that as the infill density increases, the compressive strength also increases and the variability in behavior is reduced, which favors the specimens being more consistent and robust in their mechanical properties.

**Figure 10:**
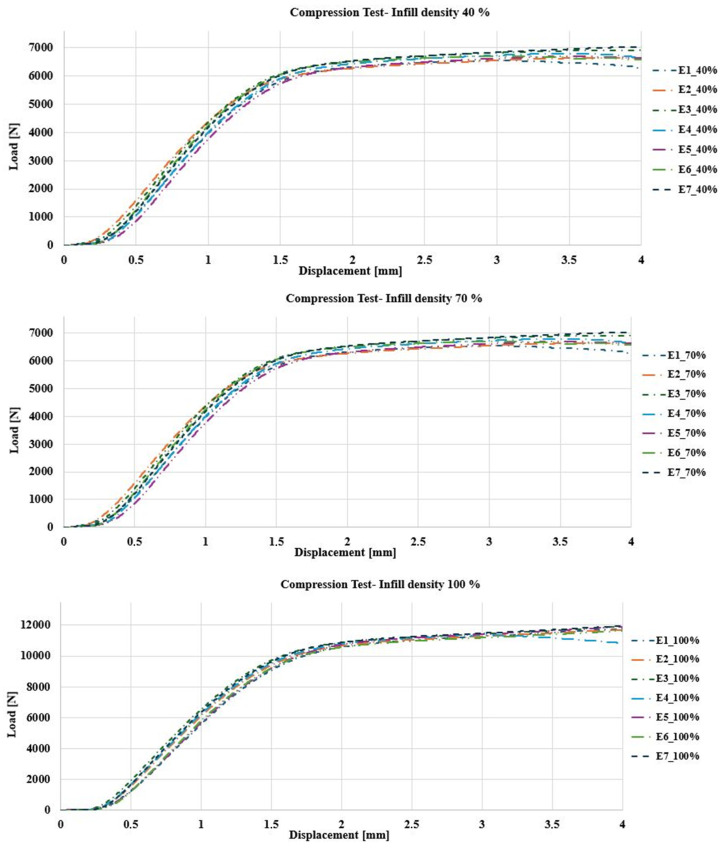
Load [N]–Displacement [mm] behavior. Compression test: specimens with 40% filling (**top**); specimens with 70% filling (**middle**); specimens with 100% filling (**bottom**).

Figure 10 shows the behavior of the load–displacement graph for each infill density.

The evaluation of PEEK specimens under compression shows that the filler density has a crucial impact on their behavior. The specimen with 40% filler showed significant deformation, with a clear narrowing in the middle part, due to the lower amount of internal material that made it more susceptible to buckling. In comparison, the 70% filled specimen showed balanced behavior with moderate deformation, reflecting a good combination of strength and energy absorption capacity.

**Figure 11:**
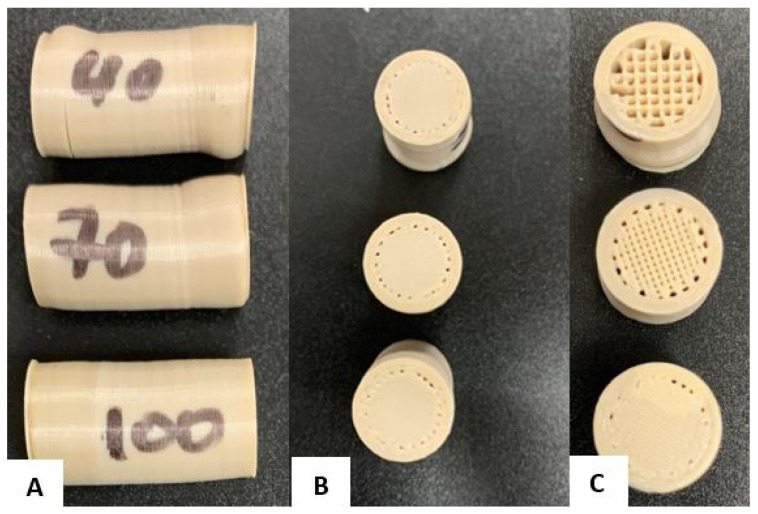
Compression test specimens: (**A**) Deformation of specimens with different filling densities (40%, 70%, and 100%) under compression. (**B**) Top view of compressed specimens showing deformation depending on infill density. (**C**) Cross-sectional view of the internal structure of the specimens after compression.

Finally, the 100% filled specimen had the least deformation, maintaining its shape as almost intact, indicating high structural rigidity and strength. This differentiated behavior suggests that, depending on the application, an appropriate infill density can be chosen to balance flexibility and strength. Figure 11 shows the results of the compression tests.

The 40% filled specimen had the lowest strength, reaching a maximum stress of 58.09MPa and showing a modulus of elasticity of 149.47MPa, which makes it more flexible and capable of deforming with an elongation of 15.76% before failure. This flexibility makes it suitable for applications where energy absorption is more important than strength. The 70% filled specimen presented a balance between strength and flexibility, withstanding a maximum stress of 78.77MPa with a modulus of elasticity of 162.06MPa and an elongation of 20.5%, which is ideal for applications that require some conformability along with strength.

Finally, the 100% filled specimen showed the highest strength, with a maximum stress of 107.53MPa, a modulus of elasticity of 147.47MPa, and an elongation of 20.29%; this shows a rigid structure, but it is still capable of deforming slightly before failing, being ideal for applications that require maximum structural stability.

These results reflect that the higher the filler density, the greater the strength and stiffness, but some of the energy absorption capacity is sacrificed, which makes each density have specific applications depending on the load and deformation requirements. Table 5 presents the summary of the average mechanical properties with the three different filler percentages. Seven specimens were tested for each percentage.

### 3.2. Statistic Analysis

This section presents the results of applying a one-factor analysis of variance (ANOVA) to the infill density in the printing of the specimens, measured at three levels: 40%, 70%, and 100%. Furthermore, a 95% confidence level was considered in analyses for the results of all destructive tests.

#### 3.2.1. ANOVA for the Bending Test

For this test, the analysis of variance showed a null *p*-value, less than 0.05. Therefore, there are statistically significant differences in the average maximum stress for the three levels of factor measurement.

The Multiple Range test showed homogeneity within two groups and non-homogeneity for the other. The obtained *p*-value for the Kruskal–Wallis test was equal to 0.001. Both tests corroborate the incidence of infill density on the observed variable.

**Figure 12:**
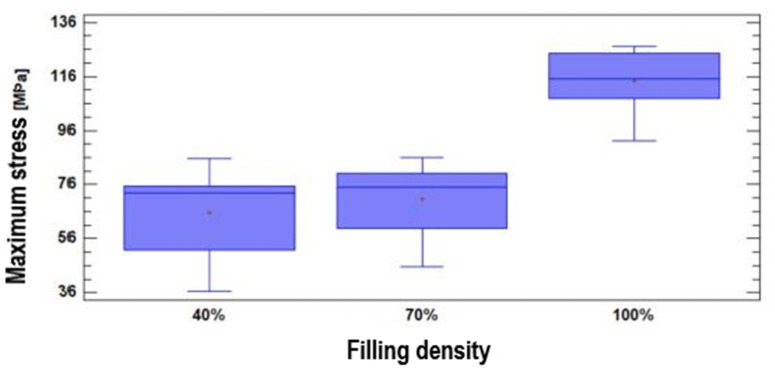
Maximum stress values according to infill density for bending testing.

In Figure 12, it can be seen that there is no significant variation in the maximum stress for the filling densities of 40% and 70%. Meanwhile, for the infill density of 100%, there is a difference in the result compared to the other two densities. It means that natural PEEK, characterized through additive manufacturing, presents a very similar flexural strength for filler densities of 40% and 70%, with values of 65.43MPa and 70.69MPa, respectively. If the infill density reaches 100%, the maximum stress of the material increases to 114.32MPa. These results show that a higher infill density significantly improves the material’s ability to resist bending stresses, which is relevant for applications requiring high mechanical strength.

Ref. [44] reports on an in vitro study of the bending of PEEK for specimens milled from discs, obtaining a bending stress of 250MPa. Also, in [8], a maximum bending stress of 163MPa is stated for PEEK OPTIMA. In the present investigation, an infill density of 100% resulted in an average maximum bending stress of 114.32MPa.

#### 3.2.2. ANOVA for Compression Test

In this case, the analysis of variance showed a null *p*-value, less than 0.05. Therefore, there are statistically significant differences in the average maximum stress for the three levels of factor measurement.

**Figure 13:**
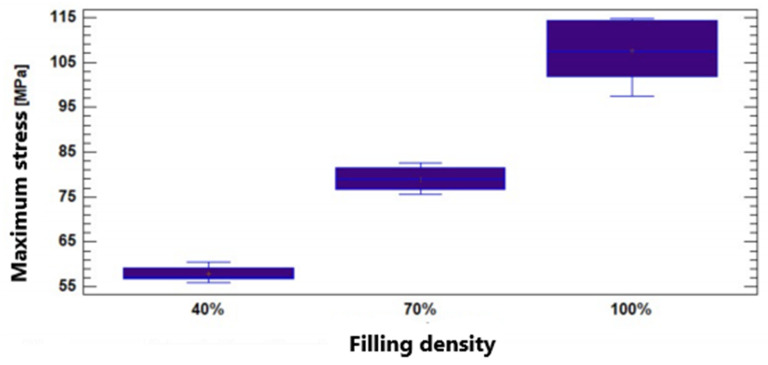
Maximum stress values according to infill density for compression testing.

The Multiple Range tests showed non-homogeneity within the three groups. The obtained *p*-value for the Kruskal–Wallis test was equal to 0.000135155. These two tests corroborate the incidence of infill density on the observed variable. In Figure 13, it can be seen that the maximum stress varies depending on the infill density. Thus, natural PEEK characterized by additive manufacturing has a compressive strength directly proportional to the filler density: 57.79MPa for 40% filler, 78.77MPa for 70% filler, and 107.53MPa for 100% filler. This shows that the increase in infill density results in a significant increase in the material’s resistance to compressive stresses.

In [45], the use of PEEK structures for implant-supported CAD-CAM prostheses is discussed. In the study, the maximum compressive stress of the implantable PEEK-OPTIMA is reported as 135MPa.

#### 3.2.3. ANOVA for the Tensile Test

The analysis of variance showed a *p*-value equal to 0.875, greater than 0.05. Therefore, there are no statistically significant differences in the average maximum stress for the three levels of factor measurement.

**Figure 14:**
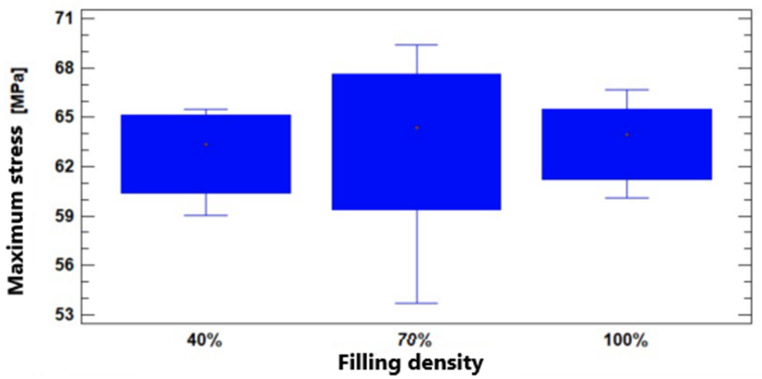
Maximum stress values according to infill density for tensile testing.

The Multiple Range test also showed homogeneity within the three groups. The obtained *p*-value for the Kruskal–Wallis test was equal to 0.261972. Both tests corroborate the non-incidence of infill density on the observed variable. In Figure 14, it can be seen that the maximum stress does not vary significantly depending on the infill density—that is, the natural PEEK characterized through additive manufacturing presents a very similar tensile strength with filler densities of 40% and 100%, with values of 63.34MPa and 63.95MPa, respectively. Meanwhile, at 70% filler, the resistance value is somewhat higher (around 64MPa).

The work [8] presents a classification of the mechanical properties of the PEEK family of polymers, and a comparison with other biomedical polymers, to determine their versatility in applications in medicine. A maximum tensile stress of 100MPa for medical grade PEEK OPTIMA is reported here, without specifying how the tests were executed. In addition, the author Hernández [46] reports on the performance of tests directly on PEEK dental prostheses, manufactured by injection, and a maximum tensile stress of 89.6MPa was obtained. It is also a very different value than that obtained for natural PEEK.

The results obtained in this study on the influence of filler density on the mechanical properties of natural PEEK processed by FDM are in line with trends observed in recent scientific literature. Furthermore, they provide new insights into how to optimize this material for specific applications in the biomedical and industrial fields.

A key finding of this study is the direct relationship between infill density and the material’s mechanical properties. This behavior is consistent with [47], where it is reported that an increase in infill density improves the strength and stiffness of 3D-printed materials but at the expense of lower ductility and energy absorption capacity. In the present study, samples with 100% infill showed the highest strength and stiffness, making them ideal for structural applications. However, an infill density of 70% demonstrated a balance between flexibility and strength, making it more suitable for critical energy absorption applications.

In [9], the mechanical properties of dental devices fabricated with PEEK using FDM are analyzed, and it is highlighted that higher filling densities are necessary for applications requiring high rigidity, such as dental and maxillofacial implants. The results of this work are consistent with this observation, as the 100% filled samples showed rigid behavior and high compressive strength.

Stepanov et al., in [48], highlight the importance of combining printing parameters, such as layer orientation and infill density, to improve the mechanical strength of PEEK. Unlike this work, which focuses exclusively on infill density, they also consider multidimensional printing configurations to optimize the material’s performance in biomedical applications. However, like them, the findings reported in this research underline that lower infill densities offer advantages in applications where greater flexibility and lower weight are required.

This research provides new insights by comparing results in terms of specific properties. For example, ANOVA statistical analysis showed significant differences in flexural strength between 70% and 100% filler densities, while no relevant differences were found in tensile strength for different densities—that is, filler density has a greater impact on properties that depend on deformation and internal load distribution. This finding extends the conclusions of [49].

In practical applications, the results obtained could guide the design of specific devices, such as the following:

- Orthopedic Prosthetics: configurations with 100% fill density are ideal for fixation plates requiring rigidity and dimensional stability.
- Dental implants: densities of 70% fill are more appropriate for applications that balance strength and energy absorption, such as maxillofacial prostheses.
- Lightweight medical devices: temporary devices, such as surgical guides, can be manufactured with 40% infill densities, optimizing weight and reducing production times.

## 4. Conclusions

The results on maximum stresses obtained in this research differ from the values reported in the revised literature for each type of test. These differences could be related to the fact that the tests were developed following standards for working with thermoplastic polymers and normalized specimens, factors that are not specified in the cited sources.

If a sterilization process is implemented for natural PEEK, it could also be a raw material for printing personalized medical devices. This would be feasible when the following requirements are met:

- If a medical device needs to withstand small bending forces, it should be manufactured with an infill density ranging between 40% and 70%. An infill density of 100% is recommended for devices undergoing significant bending.
- If the maximum tensile stresses are not required to exceed 64MPa, it would be sufficient to manufacture it with an infill density of 40%.
- If the medical device is subjected to compression forces, the maximum load it must support should be estimated to establish an appropriate infill density.

The present research could continue in several directions, among which are the following: Analyzing the mechanical behavior of natural PEEK for filler densities different from those considered. Analyzing the mechanical behavior of natural PEEK against impact. Performing destructive testing on medical grade PEEK; according to current standards, an effective mechanical comparison is possible with the behavior obtained for industrial grade natural PEEK. Performing destructive testing directly on implantable medical devices made of natural PEEK.

This study has provided a detailed understanding of how infill density influences the mechanical properties of FDM-processed natural PEEK. However, to optimize the design of medical devices and industrial components, future research evaluating additional infill percentages, such as 50% and 85%, is essential to identify configurations that balance strength and flexibility. Furthermore, it is important to analyze the impact of different infill patterns on stress distribution and the overall mechanical behavior of the printed parts.

It is also recommended that additional printing parameters, such as layer orientation and printing speed, be investigated to determine how they affect the final properties of the material. This research will allow the development of better guidelines for additive manufacturing with PEEK adapted to the particular needs of each application.

## Figures and Tables

**Table 1:** Printing parameters for natural PEEK (according to the filament manufacturer’s specifications).

Parameters	Fixed Value
Nozzle diameter (mm)	0.5
Filament diameter (mm)	1.75
Extruder temperature (°C)	445
Bed temperature (°C)	70
Chamber temperature (°C)	90
First layer height (mm)	0.2
Brim size (mm)	2.5
Max volumetric speed (mm s^−1^)	45

**Table 2:** Properties of the natural PEEK ^1^ filament at 23°C.

Mechanical Properties		
Property	Amount	Normative
Tensile strength	98MPa	ISO 527 [12]
Elongation resistance	45%	ISO 527 [12]
Young’s modulus	4000MPa	ISO 527 [12]
Impact resistance	7kJ m^2^	ISO 179-1eU [34]
Thermal properties		
Melting temperature	343°C	DIN 53765 [35]
Glass transition temperature	143°C	DIN 53765 [35]
Decomposition temperature	550°C	

^1^ The properties of PEEK were taken from https://apiumtec.com/download/apium-peek-450-datasheet, accessed on 19 September 2024.

**Table 3:** Average maximum stresses in tensile tests.

Specimen	Infill Density	Maximum Stress	Elasticity Modulus	Yield Stress
	(%)	(MPa)	(MPa)	(MPa)
1–7	40	63.44±2.60	2119.04±68.09	58.99±1.09
8–14	70	64.4±5.70	2294.22±57.86	53.70±10.06
15–21	100	63.96±2.39	2436.67±44.60	49.05±11.14

**Table 4:** Average maximum stresses in bending tests.

Specimen	Infill Density	Maximum Stress	Elasticity Modulus
	(%)	(MPa)	(MPa)
1–7	40	65.43±16.65	2134.71±603.37
8–14	70	70.69±13.99	2676.21±759.70
15–21	100	114.32±11.95	2773.73±243.23

**Table 5:** Average maximum stresses in compression tests.

Specimen	Infill Density	Maximum Stress	Elasticity Modulus
	**(%)**	**(MPa)**	**(MPa)**
1–7	40	57.79±1.57	142.05±30.35
8–14	70	78.77±2.58	162.06±42.78
15–21	100	107.53±6.29	147.47±58.36

## Data Availability

The original contributions presented in this study are included in the article.
